# Merocel versus Nasopore for Nasal Packing: A Meta-Analysis of Randomized Controlled Trials

**DOI:** 10.1371/journal.pone.0093959

**Published:** 2014-04-07

**Authors:** Jianzhang Wang, Changping Cai, Shili Wang

**Affiliations:** Department of Otolaryngology, Rui Jin Hospital, Shanghai Jiao Tong University School of Medicine, Shanghai, China; Harvard Medical School, United States of America

## Abstract

**Objective:**

To compare the clinical outcomes, including efficacy and complications, of Merocel versus Nasopore as a nasal packing material after nasal surgery.

**Methods:**

Relevant randomized controlled trials were identified from electronic databases (The Cochrane Library, PubMed, EMBASE, China National Knowledge Infrastructure and Chinese Biomedical Database). Conference proceedings and references from identified trials and review articles were also searched. Outcome measures were pain during nasal packing, pain and bleeding upon packing removal, pressure sensation, nasal blockage, formation of synechiae, mucosal healing, and patients' general satisfaction.

**Results:**

Seven randomized controlled trials met criteria for analysis. Compared with Merocel, Nasopore significantly reduced patients' subjective symptoms including *in situ* pain (pain experienced while packing is in place), nasal pressure, pain and bleeding during packing removal, and increased patients' general satisfaction with nasal packing. There were no significant differences in nasal obstruction, adhesion and mucosal healing between the Merocel and Nasopore groups.

**Conclusions:**

Preliminary evidence suggests that Nasopore may be superior to Merocel as a nasal packing material with regard to *in situ* pain, pain and bleeding upon removal, pressure, and general satisfaction and does not differ from Merocel in terms of nasal obstruction, tissue adhesion, and long-term mucosal healing.

## Introduction

Nasal packing is commonly used to control bleeding following operative procedures to the nose, including functional endoscopic sinus surgery (FESS), septoplasty, and conchotomy. It is also used to prevent middle turbinate lateralization, synechiae formation, and restenosis after FESS [1] and has been reported to stabilize the remaining cartilaginous septum internally, prevent complications such as septal hematoma and formation of synechiae, and to minimize the persistence or recurrence of septal deviation after septoplasty [2]. However, nasal packing has some inherent disadvantages, such as causing pain and bleeding and contributing to nasal mucosal damage, septal perforation, allergic reaction, sleep respiratory disturbance and decreased arterial oxygen saturation during sleep [3]. Furthermore, patients often consider packing removal to be the most unpleasant experience of their operations [4], [5]. Attempts have been made to produce materials that will address these problems, including removable and absorbable packing, and the variety of nasal packing materials has greatly increased in recent years.

The type of packing chosen by a surgeon is usually determined by habit, inherited practice, or departmental provision, and the superiority of nonabsorbable versus dissolvable nasal packing has been widely debated. Both materials can be used to control bleeding following nasal surgery and each has its own characteristics. Merocel (Medtronic Inc., Minneapolis, MN, USA), one of the most common nonabsorbable nasal packing materials, is a compressed, dehydrated sponge composed of hydroxylated polyvinyl acetate that can increase in size within the nasal cavity and compress a bleeding vessel through rehydration with normal saline. Because it is a nonabsorbable solid, disadvantages may include pain and bleeding upon removal, nasal obstruction, and mucosal edema. Nasopore (Polyganics, Groningen, The Netherlands), one of the most commonly used dissolvable materials, is a bioresorbable material produced using a freeze-drying process. It consists of fully synthetic biodegradable, fragmenting foam that absorbs water while supporting the surrounding tissue and providing pressure against bleeding vessels in the nasal cavity. It starts to dissolve within days and can be suctioned from the nasal cavity after several days.

Although many studies have been carried out comparing Merocel and Nasopore as nasal packing materials with respect to subjective symptoms and clinical efficacy, there is still no consensus as to which one is better. Two trials have reported that Nasopore packing caused significantly less pain and bleeding during removal than did Merocel packing [6], [7]; another study showed little difference in nasal symptoms between the two packing materials 5 days after surgery [8], and still another reported that Nasopore was a significant factor in the formation of excessive granulation tissue 3–4 weeks after FESS [9]. To address this issue, we performed a meta-analysis that included all available data from randomized controlled trials (RCTs) that compared Merocel with Nasopore as a packing material at the end of nasal surgery with regard to the subjective severity of the symptoms.

## Methods

This meta-analysis was guided by the Preferred Reporting Items for Systematic Reviews and Meta-analysis (PRISMA) statement [10]. No systematic review or meta-analysis concerned on this subject was found.

### Literature Search

Study selection was systematically performed in the Cochrane Central Register of Controlled Trials (CENTRAL), PubMed, EMBASE, China National Knowledge Infrastructure (CNKI) and Chinese Biomedical Database (CBM) from their inception until March 2013 and updated in September 2013. The type of language was not restricted. Search keywords were as follows: “Merocel”, “polyvinyl acetate”, “Nasopore”, “synthetic polyurethane foam”, “polyurethane foam”, “absorbable packing”, “nasal packing”, “nasal packs”, “nasal dressings”, “nasal tampon”, “nasal surgery”, “intranasal surgery”, “nasal surgical procedure”. To identify potentially eligible studies, we manually searched conference proceedings and references from identified trials and review articles. Original data were requested by directly contacting authors if necessary.

### Assessing Eligibility for Inclusion

All RCTs were retrieved that compared Merocel with Nasopore in patients undergoing post-operation of nose including FESS, septoplasty, conchotomy, or a combination. Properly randomized trials were included if 1) at least one of the outcome measures mentioned below was reported; 2) the data presented could be applied in this meta-analysis, or the original information could be obtained by contacting the corresponding author. The studies were excluded if 1) patients had a history of bleeding disorder, aspirin intolerance, asthma, allergy, or systemic diseases; 2) all data were presented as the medians and/or ranges, and the original information could not be obtained from the author.

### Data Extraction

A form was designed to extract data from each included study by two researchers (JW and SW) independently, and disagreements were resolved with a third reviewer (CC). The following information was extracted: the first author's name, year of publication, study method, treatment protocol, sample size, duration of follow-up, inclusion/exclusion criteria, and relevant outcome data.

### Outcome Measures

The primary outcome measures in this study included in situ pain, pain and bleeding upon packing removal, pressure sensation, formation of synechiae, patients' general satisfaction with nasal packing. The secondary outcome measures included nasal blockage, mucosal healing in short or long terms. Intention-to-treat (ITT) analysis was used to record clinical outcomes if needed.

### Quality Assessment

The quality of the studies was evaluated using the Cochrane handbook 5.1.0 recommended standard: random sequence generation, allocation concealment, blinding, incomplete outcome data, selective reporting, and other biases. Grading of Recommendations Assessment, Development and Evaluation (GRADE) Version 3.6 software was utilized to evaluate the evidence levels of the outcomes which were classified into four levels: high, moderate, low, and very low. Any discrepancy was resolved in consultation with the third author.

### Statistical Analysis

Review Manager Version 5.2 software recommended by Cochrane Collaboration was used in this study. The mean difference (MD) or standard mean difference (SMD) with its 95% confidence interval (CI) for continuous variable was computed and relative risk (RR) with corresponding 95% CI for dichotomous outcome data. Statistical significance was p≤0.05. Cochrane's Q test and I^2^ statistics were applied to assessing statistical heterogeneity among studies. A fixed-effects model was used when the level of heterogeneity was acceptable (p>0.1, or p≤0.1 but I^2^≤50%) and a random-effects model for the significant heterogeneity (p≤0.1, I^2^>50%).

## Results

### Search Results

**Figure 1:**
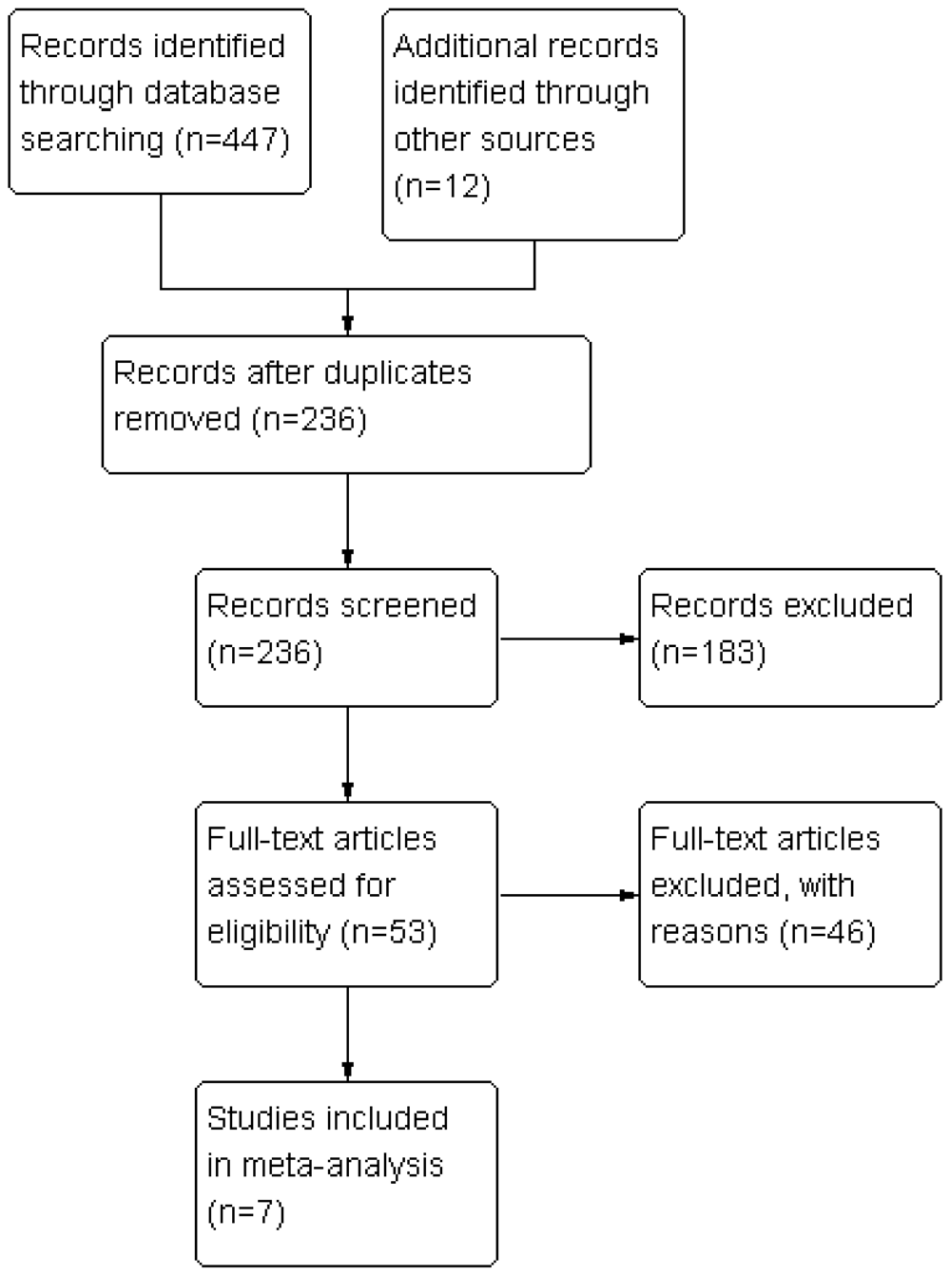
Flow diagram of study identification, inclusion, and exclusion.

The procedure for study selection is shown in Figure 1. A total of 459 potentially relevant articles were identified from the initial search; however, most were retrospective studies, review articles, non-randomized studies, case reports, or not relevant to the aim of the present study, or provided irrelevant data. Ultimately, seven trials [6]–[8], [11]–[14] were identified as eligible, all of which were RCTs published from 2009 to 2013, and consensus on study selection was reached by discussion among the researchers. Characteristics of eligible trials are summarized in Table 1.

**Table 1:** Individual studies.

Hu 2012
***Methods***: RCT (no-blind)
***Participants***: 92 patients with Chronic Rhinosinusitis (CRS) or nasal polyps, either gender, ages 17 to 67, who underwent FESS.
***Interventions***: Nasopore or Merocel was packed in both nasal cavities after FESS.
***Outcomes***: In situ pain, bleeding on packing removal, nasal synechia.
Kim 2011
***Methods***: RCT (single-blind)
***Participants***: 64 patients, either gender, ages 19 to 64, detected by paranasal sinus computed tomography and undergoing only septoplasty without any paranasal sinus problems. Patients were ineligible if they had a history of previous septoplasty, aspirin intolerance, asthma, allergy, or systemic diseases.
***Interventions***: Nasopore or Merocel was packed in both nasal cavities after septoplasty.
***Outcomes***: Pain, pressure, nasal obstruction, dysphagia, bleeding on packing removal and general satisfaction.
Kim 2013
***Methods***: RCT (single-blind)
***Participants***: 70 patients, ages 5 to 14, who underwent bilateral conchotomies.
***Interventions***: Nasopore versus Merocel nasal packing.
***Outcomes***: Headaches during nasal packing, pain and bleeding upon removal of the packing, bleeding after discharge.
Lu 2013
***Methods***: RCT (no-blind)
***Participants***: 160 patients undergoing septoplasty for nasal respiratory impairment. Patients would be excluded if they had a history of hypertension, diabetes mellitus, heart diseases or other surgical contraindication. They still should not suffer from headache, dysphagia and so on.
***Interventions***: Nasopore or Merocel was packed in both nasal cavities after septoplasty.
***Outcomes***: In situ pain, pressure, nasal fullness, fever, pain and bleeding on packing removal, mucosal healing 1 and 4 weeks postoperatively, complications of nasal septum such as perforation and hematoma.
Qian 2013
***Methods***: RCT (no-blind)
***Participants***: 98 patients, either gender, ages 20 to 76, who underwent FESS.
***Interventions***: Nasopore or Merocel was applied for postoperative packing.
***Outcomes***: Headache, fever, pain and bleeding upon removal of the packing, nasal adhesion.
Shoman 2009
***Methods***: RCT (double-blind)
***Participants***: 30 patients undergoing FESS, who met the criteria including age≥18 years, bilateral acute recurrentrhinosinusitis (ARRS) or CRS, and a Lund-MacKay computed tomographic (CT) scan score difference of 2 or less between the two nasal cavities. Patients with bleeding disorder, unilateral disease and significant difference in disease status between the left and right sides (Lund-MacKay score difference>2) were ineligible.
***Interventions***: All patients received Nasopore packing on one nasal cavity and Merocel on the other after FESS.
***Outcomes***: Subjective assessment including pain, pressure sensation, nasal blockage, swelling, bleeding during nasal packing, pain and bleeding on packing removal, mucosal healing 1 and 3 months postoperatively.
Yilmaz 2013
***Methods***: RCT (no-blind)
***Participants***: 68 patients, who underwent septoplasty for nasal respiratory impairment caused by septal deviation. Patients were ineligible if they had paranasal sinus pathologies or systemic disorders.
***Interventions***: Nasopore, silicone intranasal splint with integral airway or Merocel was applied for postoperative packing.
***Outcomes***: Pain, pressure, nasal fullness, pain and bleeding on removal, and general satisfaction.

### Quality Assessment

**Figure 2:**
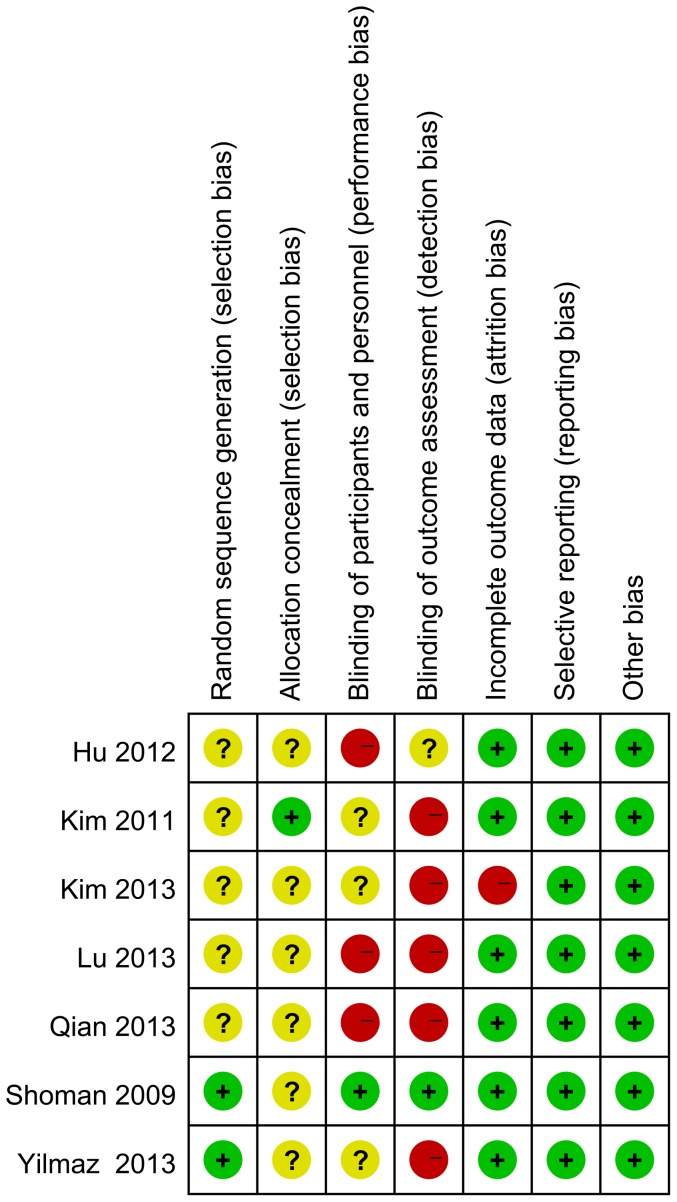
Risk of bias summary of included studies summary.

One study indicated that a block-randomization method was applied [7] and another study stated groups were randomized by a coin toss [8], whereas the remainder did not report the details of random-sequence generation. One trial reported allocation concealment with a sealed envelope [6], but concealment of allocation was an undefined risk in the other studies because it was not reported. Double blinding method was applied in one study [8], single blinding of patients in two [6], [12], and unmasked designation in one [7], blinding was not reported in the remaining three [11], [13], [14]. Funding biases were not evident in any of the studies, and they did not have baseline imbalances. Attrition bias existed and was assessed in three studies [7], [8], [12]. The above risks of bias are summarized in Figure 2.

Outcomes analyzed and assessed by GRADEprofiler presented some limitations in study design or execution and inconsistency in results but no obvious indirectness or imprecision evident. Funnel-plot analysis was not conducted to evaluate the risk of publication bias because asymmetry could not be reliably assessed with only seven RCTs and any no unpublished negative studies were found. Given this, the quality of each outcome is shown in Table 2.

**Table 2:** Quality assessment of included studies by GRADE analysis.

Outcome measure	Subjects (studies)	Risk of bias	Inconsistency	Indirectness	Imprecision	Other considerations	Overall quality^5^	Importance
**In situ pain**	426(5)	serious^1^ ^,2,3^	Serious^4^	no serious indirectness	no serious imprecision	undetected	+/+/−/−/; low	critical
**Pain on removal**	502(6)	serious^1^ ^,2,3^	Serious^4^	no serious indirectness	no serious imprecision	undetected	+/+/−/−/; low	critical
**Bleeding on removal**	636(6)	serious^1^ ^,2,3^	Serious^4^	no serious indirectness	no serious imprecision	undetected	+/+/−/−/; low	critical
**Nasal obstruction**	334(4)	serious^1^ ^,2,3^	Serious^4^	no serious indirectness	no serious imprecision	undetected	+/+/−/−/; low	important
**Nasal pressure**	334(4)	serious^1^ ^,2,3^	Serious^4^	no serious indirectness	no serious imprecision	undetected	+/+/−/−/; low	critical
**Tissue Adhesion**	430(3)	serious^1^ ^,2,3^	no serious inconsistency	no serious indirectness	no serious imprecision	undetected	+/+/+/−/; moderate	critical
**Mucosal healing at 1-week**	160(1)	serious^1^ ^,2,3^	no serious inconsistency	no serious indirectness	no serious imprecision	undetected	+/+/+/−/; moderate	important
**Mucosal healing at 4-week**	160(1)	serious^1^ ^,2,3^	no serious inconsistency	no serious indirectness	no serious imprecision	undetected	+/+/+/−/; moderate	important
**Mucosal healing at 1-month**	60(1)	Serious^3^	no serious inconsistency	no serious indirectness	no serious imprecision	undetected	+/+/+/−/; moderate	important
**Mucosal healing at 3-month**	60(1)	Serious^3^	no serious inconsistency	no serious indirectness	no serious imprecision	undetected	+/+/+/−/; moderate	important
**General satisfaction**	107(2)	serious^1^ ^,2,3^	Serious^4^	no serious indirectness	no serious imprecision	undetected	+/+/−/−/; low	critical

1 Insufficient information about the sequence generation process. ^2^ Insufficient information about the allocation concealment. ^3^ Blinding was not adequate. ^4^ I^2^>50%. ^5^ High quality: further research is very unlikely to change our confidence in the estimate of effect; moderate quality: further research is likely to have an important impact on our confidence in the estimate of effect and m ay change the estimate; low quality: further research is very likely to have an important impact on our confidence in the estimate of effect and is likely to change the estimate; very low quality: we are very uncertain about the estimate.

### Outcomes and synthesis of results

#### Pain in Situ and upon Removal

**Figure 3:**
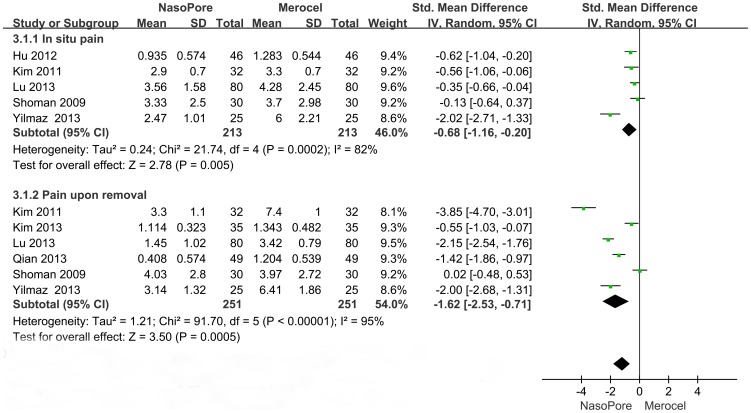
Forest plots of SMD and 95% CI for in situ pain and pain upon removal.

Meta-analysis of in situ pain showed that the pain score in the Nasopore group was lower than in the Merocel group (SMD, −0.68; 95% CI, −1.16 to −0.20; p = 0.005). Statistical heterogeneity was observed among the studies (chi square  = 21.74, I^2^ = 82%; p = 0.0002). Meta-analysis of pain on removal showed significant differences between the two groups (SMD, −1.62; 95% CI, −2.53 to −0.71; p = 0.0005). There was statistical heterogeneity among the trials (chi square  = 91.70, I^2^ = 95%; p<0.00001). Compared with Merocel, Nasopore significantly reduced pain in situ and upon removal (Figure 3).

#### Bleeding upon Removal

**Figure 4:**
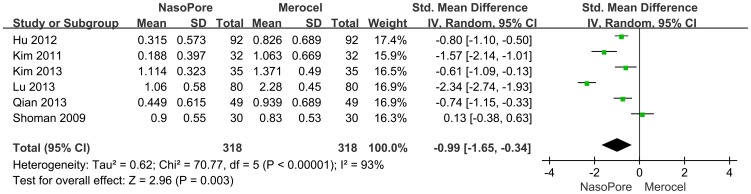
Forest plots of SMD and 95% CI for bleeding upon removal.

Six studies were included in the meta-analysis. As shown in Figure 4, there is a significant difference in bleeding upon removal between the two groups (SMD, −0.99; 95% CI, −1.65 to −0.34; p = 0.003). Statistical heterogeneity existed among the studies (chi square  = 70.77, I^2^ = 93%; p<0.00001). Patients in the Nasopore group received more clinical benefit and better control of bleeding than did the Merocel group.

#### Nasal Obstruction and Pressure

**Figure 5:**
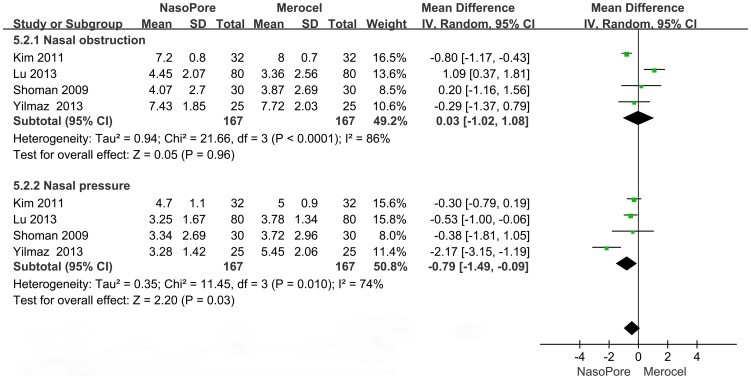
Forest plots of MD and 95% CI for nasal obstruction and pressure.

Both nasal obstruction and pressure were graded using a visual analog scale (VAS) in the four included studies. No significant difference was observed in nasal obstruction during packing between the two groups (MD, 0.03; 95% CI, −1.02 to 1.08; p = 0.96), but statistical heterogeneity was observed (chi square  = 21.66, I^2^ = 86%; p<0.0001). There was a significant between-group difference in nasal pressure (MD, −0.79; 95% CI, −1.49 to −0.09; p = 0.03), and there was statistical heterogeneity (chi square = 11.45, I^2^ = 74%; p = 0.01) (Figure 5). Thus, there was no significant difference with regard to nasal blockage, but there was less nasal cavity pressure in the Nasopore group than in the Merocel group.

#### Tissue Adhesion

**Figure 6:**
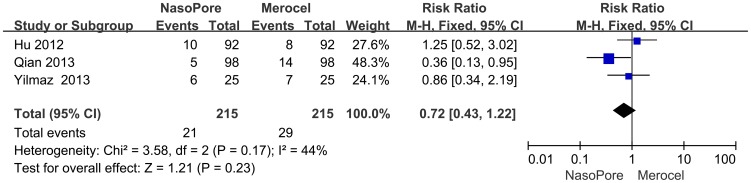
Forest plots of RR and 95% CI for nasal tissue adhesion.

Adhesion was reported in three studies. According to Figure 6, analysis of these studies suggested that there was no significant between-group difference in adhesion caused by nasal packing (RR, 0.72; 95% CI, 0.43 to 1.22; p = 0.23), and there was no obvious statistical heterogeneity (chi square  = 3.58, I^2^ = 44%; p = 0.17).

#### Mucosal Healing

**Figure 7:**
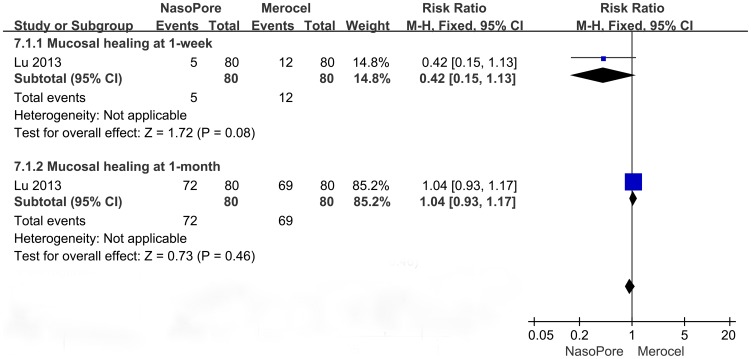
Forest plots of RR and 95% CI for mucosal healing at 1-week and 1-month.

**Figure 8:**
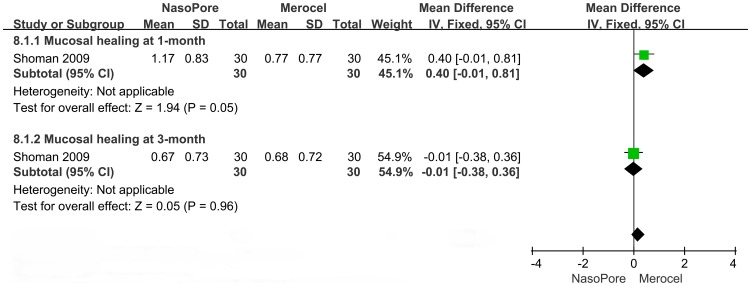
Forest plots of MD and 95% CI for mucosal healing at 1-month and 3-month.

Only two studies supplied information about mucosal healing between groups: one reported morbidity in each group and the other graded the degree of sinonasal mucosa using a symptom scale. As shown in Figure 7, there was no significant between-group difference in the effect of packing material on mucosal healing rate at the first or fourth week after the operation (RR, 0.42 to 1.04; 95% CI, 0.15 to 1.17; p = 0.08 to 0.46). As shown in Figure 8, mucosal grading at the 1-month-postoperative visit was better for the Merocel group than for the Nasopore group (MD, 0.4; 95% CI, −0.01 to 0.81; p = 0.05), but mucosal healing reassessed at 3 months was not significantly different between groups (MD, −0.01; 95% CI, −0.38 to 0.36; p = 0.96).

#### General Satisfaction

**Figure 9:**
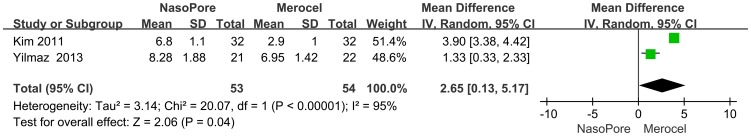
Forest plots of MD and 95% CI for general satisfaction.

General satisfaction scores with regard to nasal packing material were assessed using VAS scores in two studies. Statistical heterogeneity was noted (chi square  = 20.07, I^2^ = 95%; p<0.00001). As shown in Figure 9, there were significant differences in general satisfaction between groups (MD, 2.65; 95% CI, 0.13 to 5.17; p = 0.04); therefore, general satisfaction score and willingness to reuse the product was much higher for the Nasopore group than for the Merocel group.

## Discussion

Nasal packing was first described in the otorhinolaryngologic literature in 1951 [15], and since then it has been designed to repress mucosal bleeding and improve wound healing postoperatively. Unfortunately, pain upon its removal has been described by patients as the most unpleasant aspect of the surgical experience [4], [5] and it can increase postoperative morbidity including infection [16] and sleep-disordered breathing [17] and so on. Some researchers have even reported that nasal packing did not have a definite advantage in improving nasal airways after nasal surgery and have advocated no packing of the middle meatus thereby preventing packing complications and reducing economic burden [18]–[20]. However, many others believe that it is still necessary. For instance, microporous polysaccharide hemospheres effectively reduced postoperative bleeding during the early recovery period after FESS in a prospective RCT [21], and chitosan gel controlled-release system has been shown to contribute to wound healing and reduce adhesion formation after FESS in a sheep model [22]. Controversy still exists about whether to pack or not. These inconsistencies are multifactorial and may derive from differences in dressing composition, surgical technique, evaluation methodology, and perioperative management (debridement, nasal irrigation, topical corticosteroids, and/or antibiotics). Moreover, nasal packing is usually required for patients with hypertension, diabetes mellitus, or severe inflammatory response. Most surgeons today still consider nasal packing to be the traditional strategy of controlling ongoing bleeding after nasal surgery and in epistaxis [23], so a suitable nasal packing material needs to be developed.

The ideal nasal packing should be able to control bleeding and minimize pain and discomfort, damage to the nasal mucous membrane, and tissue reaction [24], [25]. Many nasal packing products are available, in both removable- and absorbable- pack forms. Merocel is one of the most popular nasal dressings and possesses many advantages, such as low price, ease of manipulation, excellent wet-state elasticity and sufficient support, but the severe pain and bleeding upon removal that patients experience is its major drawback [4], [5], [26]–[28]. In addition, removable nasal packs generate other complications such as pack dislodgement, septal perforation, toxic shock syndrome, obstructive sleep apnea secondary to nasal obstruction, and even death [1], [29]. Given this, ongoing attempts have been carried out to develop absorbable biomaterials that can control bleeding effectively, provide middle turbinate support, and promote wound healing yet not require removal. Nasopore, one of the most prevalent absorbable agents has advantages including biodegradability, ease of manipulation, and no need for post-operative removal; however it has been reported that Nasopore tended to induce excessive granulation tissue formation during the early stages of wound healing postoperatively [9], results that conflicted with those of previous trials. Therefore, this meta-analysis was conducted and the pooled results of each outcome were concisely described from primary to secondary outcome measures.

As mentioned, pain upon removal was the most important outcome. Of the six included RCTs, five resulted in significantly reduced pain and patient discomfort during removal of Nasopore compared with Merocel and meta-analysis of pain on removal supported this conclusion. Pooled results of in situ pain were also obtained. Because patients were under anesthesia when the nasal packs were placed after surgery, insertion pain was not described. Only one original study concluded that Nasopore had a lower nasal pressure score than Merocel while three trials concluded that the difference between the two products was not statistically significant. However, the meta-analysis suggested that Nasopore was superior to Merocel in terms of pressure. There was no significant difference in nasal adhesion between these two groups. Meta-analysis of general satisfaction demonstrated that Nasopore groups had higher satisfaction scores than Merocel group. The superiority of Nasopore over Merocel demonstrated in these primary outcome measures allows an evidence-based decision.

Nasal blockage experienced within days after surgery was classified as a secondary outcome because breathing through the mouth is possible. The pooled results of four RCTs suggested no significant difference in nasal blockage between the two groups. Mucosal healing is a less obvious though important outcome after FESS and is affected not only by packing material but also by many other factors, such as sinus cavity debridement, topical and systemic steroids, nasal irrigation, and oral antibiotics [27]. Therefore, it was categorized as a secondary outcome and the meta-analysis showed that there was no statistical significance in the two mucosal grading scores between groups over the long term.

This meta-analysis of RCTs compared postoperative subjective severity of symptoms and clinical efficacy of Merocel with those of Nasopore and suggests that Nasopore may be of greater benefit to patients than Merocel as a nasal packing material. In situ pain, pain on removal, bleeding, and pressure were significantly lower in patients treated with Nasopore packs, and general satisfaction and willingness to use the product again were higher in the Nasopore group than in the Merocel group. Moreover, nasal obstruction, the occurrence of nasal adhesion, and long-term mucosal healing were not statistically different between the two groups. This study may therefore provide surgeons with an evidence-based strategy for choosing a type of nasal dressing.

Several potential limitations of this meta-analysis should be considered: 1) There were only seven RCTs included and early attrition existed in three trials, and ITT analysis was used to extract the lost data, so the results should be interpreted with caution. 2) Pooled results revealed that the possibility of significant heterogeneity. This may be from the inclusion of trials differing in aspects including methodology, patient and treatment selection, assessment method, follow-up duration, study size and year, and outcome variables. Therefore, a random-effects model was employed to conduct a conservative estimate in the present study. 3) Because the results of a funnel-plot analysis based on a limited number of studies were not reliable, we did not analyze funnel plots to show risks of publication bias, which may have gone undetected.

## Conclusions

To date there has not been clarity as to which of the two fairly popular nasal dressings–Merocel, a nonabsorbable dressing, or Nasopore, an absorbable one–is superior. This meta-analysis provides preliminary evidence that Nasopore is superior to Merocel with regard to pain upon removal, bleeding, in situ pain, pressure, and general satisfaction and equal to Merocel with regard to nasal obstruction, tissue adhesion, and mucosal healing. This evidence may be useful to otolaryngologists choosing nasal packs after FESS, septoplasty, and conchotomy and to emergency physicians choosing nasal packs to control epistaxis. Nevertheless, the evidence levels for clinical measures were low and application of results in guiding practice should be done with caution because of the relatively small sample size, high degrees of heterogeneity, and high risk of bias. Our meta-analysis indicates that this subject requires the conduct of more RCTs with high methodological quality to resolve problems with aspects such as random-sequence generation; allocation concealment; incomplete outcome data; selective reporting; key-point reporting; and blinding of participants, personnel and outcome assessment, and suggests that RCT reporting should be guided by the Consolidated Standards of Reporting Trials (CONSORT) statement [30].

## Supporting Information

Checklist S1 **PRISMA checklist.**
(DOC)
